# Endoplasmic Reticulum (ER) Stress Response and Its Physiological Roles in Plants

**DOI:** 10.3390/ijms14048188

**Published:** 2013-04-15

**Authors:** Yan Deng, Renu Srivastava, Stephen H. Howell

**Affiliations:** Plant Sciences Institute and Department of Genetics, Development and Cell Biology, Iowa State University, Ames, IA 50011, USA; E-Mails: ydeng@iastate.edu (Y.D.); renu@iastate.edu (R.S.)

**Keywords:** endoplasmic reticulum stress, unfolded protein response (UPR), endoplasmic reticulum quality control (ERQC), endoplasmic reticulum associated degradation (ERAD), autophagy, cell death

## Abstract

The endoplasmic reticulum (ER) stress response is a highly conserved mechanism that results from the accumulation of unfolded or misfolded proteins in the ER. The response plays an important role in allowing plants to sense and respond to adverse environmental conditions, such as heat stress, salt stress and pathogen infection. Since the ER is a well-controlled microenvironment for proper protein synthesis and folding, it is highly susceptible to stress conditions. Accumulation of unfolded or misfolded proteins activates a signaling pathway, called the unfolded protein response (UPR), which acts to relieve ER stress and, if unsuccessful, leads to cell death. Plants have two arms of the UPR signaling pathway, an arm involving the proteolytic processing of membrane-associated basic leucine zipper domain (bZIP) transcription factors and an arm involving RNA splicing factor, IRE1, and its mRNA target. These signaling pathways play an important role in determining the cell’s fate in response to stress conditions.

## 1. Introduction

The ER is the gateway to the secretory pathway in plant cells. About one-third of all proteins are secreted or are membrane proteins that are folded and assembled in the endoplasmic reticulum (ER). Proper folding is important for transport and function of these proteins. Plants maintain a balance between protein folding demand and folding capacity. When that balance is disturbed by conditions, such as environmental stress, unfolded and misfolded proteins accumulate in the ER, causing “ER stress”, a condition, which can do damage to cells. To deal with this problem, cells are equipped with a sophisticated ER quality control system (ERQC). The accumulation of unfolded or misfolded proteins activates the unfolded protein response (UPR), which upregulates the production of factors that promote protein folding and/or remove unfolded/misfolded proteins from ER through ER associated degradation (ERAD). Most of our knowledge about this response comes from studies in yeast and mammalian systems. Our current understanding about these processes in plants is still rather limited. In this review, we present a brief outline of the general principles of protein folding, ERQC, ERAD and ER stress-induced autophagy in yeast and mammals. Further, we describe the UPR mechanisms in yeast, mammals and plants and, finally, focus on the physiological roles of UPR in plants.

## 2. Protein Folding and ER Quality Control

Protein folding involves all the processes by which nascent proteins attain their native and functional conformation. Nascent proteins destined for secretion bear *N*-terminal signal peptides that interact with signal recognition particles (SRPs) [1]. SRP binding stalls translation, and the SRP-containing complex of mRNA, ribosome and nascent polypeptide is led to the ER membrane, where SRP dissociates. Translation then resumes, and the growing polypeptide is translocated into the ER lumen through the Sec61 translocon complex [1–3]. Protein folding starts immediately upon entry into the ER lumen and is aided by various molecular chaperones and enzymes. The major role of chaperones is to bind to nascent proteins and prevent their aggregation. In an unfolded state, hydrophobic regions of the protein can be surface exposed, making the proteins vulnerable to aggregation.

There are two major pathways for protein folding, *N*-glycan-dependent and *N*-glycan-independent pathways. The *N*-glycan-independent pathway involves the lumenal binding protein (BiP) or the 94-kDa glucose regulated protein (GRP94). BiP is an abundant chaperone in the ER, where it associates with the Sec61 translocon and interacts with newly synthesized polypeptides. Its interaction is aided by a co-chaperone, ERdj3, a DNA J protein, in a nucleotide-dependent manner [4]. BiP has a *N*-terminal nucleotide binding domain and a *C*-terminal substrate-binding domain, which can bind to hydrophobic patches of non-native state proteins, such as those forming β-strands buried in the protein core. ERdj3 binds directly to nascent proteins and recruits BiP. This induces BiP’s ATPase activity [4,5] and the hydrolysis of bound ATP to ADP. In the ADP-bound form, BiP binds substrates with high affinity. Exchange of ADP with ATP releases the substrate, which can then progress further through the folding process [6,7]. GRP94 is less well characterized than BiP and was found to associate with fewer newly synthesized proteins, preferring partially folded substrates [8,9]. Another glycan independent process is oxidative protein folding, which involves protein-disulfide isomerases (PDI family proteins). PDI family proteins are responsible for the formation of disulfide bonds and the exchange of bonds between cysteine residues in unfolded proteins [10].

**Figure 1:**
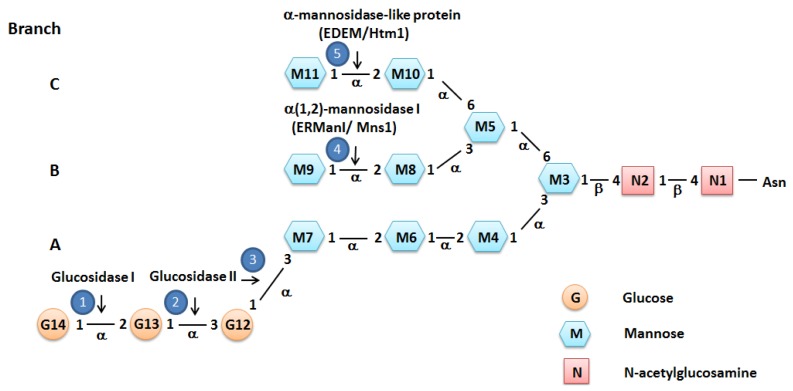
Structure of the *N*-linked oligosaccharide Glc3Man9GlcNAc2. *N*-linked oligosaccharides on glycoproteins consist of three glucoses (orange circle), nine mannoses (blue hexagon) and two *N*-acetylglucosamines (pink square) and is a branched structure with three branches, A, B and C. The numbers inside the residues indicate the order in which they are added during synthesis, and the numbers inside the dark blue circles indicate the order of their modification. The types of residue linkages and the glucosidases and mannosidase involved in modification processes are also shown.

The other folding pathway is *N*-glycan-dependent. Most secreted and membrane proteins are co- or post-translationally glycosylated at context-dependent asparagine (Asn) residues [11]. A preassembled oligosaccharide is transferred from its membrane-localized carrier, dolichyl pyrophosphate (Dol-PP), to the nascent polypeptide, catalyzed by oligosaccharide transferase (OST), an integral membrane multisubunit complex [12]. The transferred oligosaccharide is made up of three glucoses, nine mannoses and two *N*-acetylglucosamines (Glc3Man9GlcNAc2) and is a branched structure with three branches, A, B and C (Figure 1). Immediately following glycan transfer, the outermost glucose residue (G14) on branch A is removed by glucosidase I (GI), an ER membrane protein. The next step is the removal of the second glucose residue (G13) by glucosidase II (GII), an ER membrane heterodimeric enzyme [13]. The resulting monoglucosylated oligosaccharide is recognized by the lectin chaperones, calnexin (CNX) and calreticulin (CRT).

Although some functions differ in the *N*-glycan-dependent and -independent pathways, the pathways collaborate in protein folding through sequential interaction with BiP and CNX/CRT [14].

**Figure 2:**
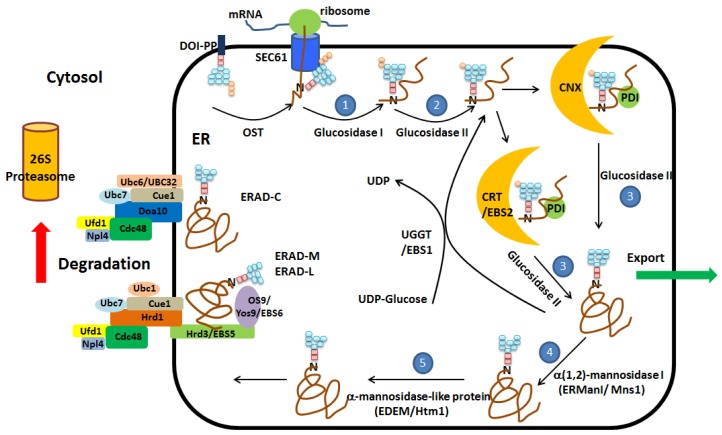
Protein folding and misfolding leading to export or endoplasmic reticulum associated degradation (ERAD). Preassembled oligosaccharides are transferred from dolichyl pyrophosphate (Dol-PP) to newly synthesized polypeptides, catalyzed by oligosaccharide transferase (OST). The two outermost glucose (Glc) residues are rapidly removed by GI and GII, allowing the nascent proteins to be picked up by the CRX or CRT folding apparatus and to fold, aided by members of the family of protein-disulfide isomerases (PDI) proteins. Removal of the last Glc residue allows properly folded proteins to be exported to Golgi, whereas misfolded proteins are reglucosylated by glycoprotein glucosyltransferase (UGGT) and re-enter the CNX/CRT folding cycle. Misfolded proteins that fail to achieve their native conformation are extracted from further CNX/CRT folding cycles by sequential removal of the outer α1,2 mannoses (Man) and recognized and ubiquitinated by Hrd1 (for ERAD-M and ERAD-L substrates) or Doa10 (for ERAD-C substrates) E3 complexes. The misfolded proteins are dislocated from the ER by the Cdc48 complex and sent to 26S proteasome for degradation. The numbers inside the blue circles indicate the order of the modification, which is consistent with the ones in Figure 1. The genes representing the *EBSs* in *Arabidopsis* are also shown.

CNX and CRT are two closely related calcium-binding chaperones in the ER that can recruit other ER chaperones and “foldases” to assist protein folding. CNX is a type I ER membrane protein, while CRT is an ER lumenal protein, which is highly homologous to CNX. There are two types of foldases, peptidyl-prolyl isomerase (PPI) and PDI family proteins. PPI catalyzes the isomerization of prolyl peptide bonds and, in doing so, accelerates protein folding. Glycoproteins are released from the CNX or CRT folding machinery by further cleavage of the last glucose residue (G12) catalyzed by GII, and the fully folded protein (Man9GlcNAc2) is subsequently transported as cargo in vesicles that traffic from the ER to the Golgi (Figure 2).

The proper folding of proteins, particularly large proteins, can be challenging, since the folding energy landscape is complex. Even under optimal conditions, protein misfolding can still happen. Under stress conditions or at specific developmental stages in which there is heavy protein secretion, the demands for protein folding in the ER can exceed its folding capacity. The resulting accumulation of unfolded/misfolded proteins produces ER stress, which can be harmful to the cells and the whole organism.

To deal with this, the ER has a sophisticated ERQC system that can detect and retain misfolded proteins for additional rounds of folding and eliminate terminally misfolded proteins through ERAD or autophagic degradation. Released glycoproteins that are not folded properly are recognized and reglucosylated (monoglucosylated) by uridine diphosphate (UDP)-glucose: glycoprotein glucosyltransferase (UGGT). The resulting monoglucosylated glycoproteins (GlcMan9GlcNAc2) are recognized and subjected to additional rounds of folding (Figure 2).

In plants, homologs of the key components of the protein folding and ERQC machinery have been identified by sequence similarity; however, the function of these proteins needs to be demonstrated. Interestingly, some conserved components of the ERQC system have been identified as ethyl methanesulfonate (EMS)-mutagenized *bri1-9* suppressors (EBSs) in *Arabidopsis*, such as EBS1/UGGT and EBS2/CRT3 [15,16]. BRI-9 protein is a structurally defective, but biochemically functional brassinosteroid (BR) receptor, which is retained in the ER by ERQC under normal conditions, while the *ebs* mutants have defects in the ERQC system and allow BRI1-9 to be transported to the plasma membrane. *Arabidopsis EBS1* encodes a homolog of mammalian UGGT, which reglucosylates the oligosaccharides on misfolded proteins and retains them in the CNX/CRT protein folding cycle, while *EBS2* encodes CRT3. In *ebs1* and *ebs2* mutants, defective UGGT or CRT fails to retain the BRI1-9 receptor in the CNX/CRT protein folding cycle and allows it to be transported to the plasma membrane.

## 3. ER Associated Degradation (ERAD)

Misfolded proteins that fail to achieve their native state are degraded by the ERAD system. Glycoproteins are extracted from the UGGT/CNX/CRT/GII cycle after removal of the outermost mannose (M9) on branch B by the ER localized α(1,2)-mannosidase I (ERManI/Mns1 in mammals/yeast) [13] (Figures 1 and 2). Compared to the other reactions, this step can be slow, allowing misfolded proteins to undergo additional rounds of protein folding. If the glycoprotein is still not properly folded after additional rounds of folding, the outermost mannose (M11) on branch C is removed by the ER degradation-enhancing α-mannosidase-like proteins (EDEMs/Htm1, in mammals/yeast) and targeted to the ERAD system (Figures 1 and 2).

ERAD involves four steps—recognition, ubiquitination, dislocation and the degradation. Based on the subcellular localization of the misfolded domain of the substrate, there are three different ERAD pathways through which a misfolded protein can be eliminated: ERAD-L for proteins with misfolded domains in the ER lumen, ERAD-M within the ER membrane and ERAD-C in the cytoplasm. In yeast, ERAD-L substrates are recognized by Hrd3 and Yos9, depending on both the folding state and glycosylation state of the misfolded protein [13]. Hrd3 (SeL1L in mammals) is an E3 ubiquitin ligase responsible for the recognition and binding of the misfolded protein based on the folding state. Yos9 (OS9/XTP3-B in mammals) is a lectin with a mannose-6-phosphate receptor homology domain that physically interacts with Hrd3/Sel1L and recognizes and binds misfolded proteins based on their glycosylation state (Man7GlcNAc2). The chaperone and foldase, BiP and PDI family protein also play a role in the recognition process possibly through selecting ERAD substrates based on the time spent in their folding cycles [17,18] (Figure 2).

Misfolded proteins are recruited to the ER membrane-embedded E3 complex for ubiquitination. Yeast has two such complexes: the Hrd1 complex, which is involved in the ubiquitination and degradation of ERAD-L and ERAD-M substrates, and the Doa10 complex, which is involved in the ubiquitination and degradation of ERAD-C substrates [19]. Hrd1 and Doa10 are E3 ligases, and both complexes contain other components, such as Ubc6 and Ubc7, ubiquitin-conjugating enzymes (E2) and Cue1, an ER membrane protein that recruits the Ubc7 to the Hrd1 and Doa10 (Figure 2). Degradation of the ubiquitinated, misfolded proteins is carried out by the 26S proteasome that is localized in the cytosol. Thus, misfolded glycoproteins have to be dislocated across the ER membrane and returned back to the cytoplasm [20]. In yeast, Cdc48 (p97 in mammals) and its cofactors, Npl4 and Ufd1, carry out the retrograde translocation of the misfolded protein from ER, presumably through channels in the Hrd1 or Doa10 complexes. Cdc48 is an AAA-ATPase family motor protein originally isolated as a cell cycle mutant in yeast.

After dislocation, the Cdc48 complex delivers misfolded proteins to the proteasome via Ufd2 and Rad23 [21,22]. Ufd2 is an E4 enzyme essential for polyubiquitin chain assembly and can bind to both Cdc48 and Rad23. Rad23 is a protein that contains an ubiquitin-associated domain and an ubiquitin-like domain, so it can bind directly to the ubiquitin chains on a substrate and also to the proteasome subunit Rpn1, serving as a bridge between the polyubiquinated substrate and the proteasome. Cdc48 can free Rad23 from Ufd2 to allow the transfer of the substrate to the proteasome for degradation [22].

In contrast to yeast and mammals, our understanding of ERAD in plants is still limited. Some of the first evidence for ERAD in plants came from studies in which an assembly-defective phaseolin, a trimeric vacuolar storage glycoprotein, was expressed in plants [23–25]. Assembly-competent phaseolin trimerizes and traffics from ER to vacuole, while the assembly-defective phaseolin is detained by its association with BiP and slowly degraded. ERAD in plants has also been demonstrated by expressing the catalytic A chain of the heterodimeric toxin ricin (RTA) in the absence of the B chain [26,27]. When expressed in tobacco protoplasts, RTA was dislocated (retrotranslocated) from ER to the cytosol, deglycosylated and degraded in a manner that was inhibited by proteasome inhibitors, clasto-lactacystin beta-lactone and MG132. It was shown that retrotranslocation of RTA requires the ATPase activity of CDC48, which is also characteristic of ERAD [28]. Similarly, when a mutated form of the barley seven-transmembrane domain mildew resistance o (MLO) protein was expressed in plants, it was also degraded by ERAD, as demonstrated by the fact that its degradation was blocked by the proteasome inhibitors and required the ATPase activity of CDC48 [29]. When the mutated form of MLO was expressed in yeast, it was also recognized as a ERAD substrate and degraded. When the mutated MLO was expressed in *hrd1* deletion yeast strains, it was stabilized, indicating that the degradation of misfolded MLO proteins depended on the Hrd1 E3 complex.

In *Arabidopsis*, orthologs of yeast Hrd1, Hrd3, Ubc6, Yos9 and Cdc48 have been identified [29–34]. Notably, orthologs of yeast Hrd3 and Yos9 have also been identified as *bri1-9* suppressors, *ebs5* and *ebs6*[30,33]. Arabidopsis mutants knocking out both *HRD1A* and *HRD1B*, homologs of yeast *Hrd1*, and *UBC32*, an ortholog of mammalian *Ubc6*, can also suppress the *bri1-9* dwarf phenotype [30,34]. All these proteins are components of HRD1 complex, which recognizes and ubiquitinates misfolded proteins. It was proposed that defective Hrd1 complexes prevent BRI1-9 protein from being eliminated by ERAD and allow it to be transported to the plasma membrane.

## 4. Autophagic Degradation

Beside the proteasome dependent ERAD system, autophagy may also be involved in the removal of ERAD substrates. Autophagy functions as a degradation system in recycling cellular contents under stress conditions. Upon induction of autophagy in mammals, a double membrane structure, called an autophagosome, engulfs targeted cellular components. The outer membrane of the autophagosome fuses with lysosomes to form autolysosomes, which degrade the inner membrane and the cargo [35]. It has been proposed that the autophagosome membrane may derive from several sources, including the plasma, ER and mitochondria membranes.

Autophagy is involved in diverse biological responses in plants, such as nutrient starvation, pathogen infection, salt and drought stresses and senescence [35]. A recent study showed that ER stress elicited by ER stress agents, tunicamycin (TM) and dithiothreitol (DTT), can also induce autophagosome formation in *Arabidopsis*. An ER membrane decorated with ribosomes was observed inside the autophagic bodies in the vacuole, likely for degradation [36], indicating that autophagy may turn over the ER membrane and its contents in response to ER stress in plants.

## 5. Unfolded Protein Response (UPR)

The UPR is activated by the accumulation of unfolded/misfolded proteins in the ER. The UPR is a homeostatic response to alleviate ER stress through transcriptional and translational events that reduce the production of secreted and membrane proteins and increase the synthesis of chaperones, foldases and other components involved in ERQC and ERAD systems. These represent cell-sparing activities, and if they fail, then programed cell death (PCD) may ensue. While the UPR signaling pathways have been mainly worked out in yeast and mammals, similar pathways have been identified in plants in recent years.

The UPR is usually induced in the laboratory by treatment with ER stress agents, such as TM or DTT. TM induces ER stress by blocking the transfer of oligosaccharides onto nascent ER proteins, while DTT is thought to generate ER stress by disrupting the redox conditions needed for the formation of disulfide bridges in proteins. It should be pointed out that while both TM and DTT result in unfolded/misfolded protein accumulation in the ER, they are simply proxies for the natural environmental conditions that elicit the UPR.

### 5.1. UPR in Yeast

Inositol requiring enzyme-1 (IRE1) was discovered as the first UPR sensor in budding yeast [37]. IRE1 is a type I, single pass ER membrane protein that contains both a kinase domain and a RNase domain on the *N*-terminal side of the protein facing the cytoplasm. Upon ER stress, IRE1 dimerizes and/or oligomerizes, clustering into foci [38,39], and undergoes trans-autophosphorylation, which activates its RNase activity. The RNase activity in yeast IRE1 catalyzes the initial step in the nonconventional, splicing of *HAC1* mRNA in a spliceosome-independent manner [37,39]. *HAC1* mRNA splicing removes a 252 b intron located at the 3′ end of the RNA. Unlike normal mRNA splicing, which occurs in the nucleus, this splicing takes place in the cytosol. After splicing, *HAC1* mRNA is ligated by a tRNA ligase, Rig1 [40]. The unspliced form of *HAC1* mRNA contains a translational inhibitor in its intron, while the spliced form encodes a basic leucine zipper domain (bZIP) transcription factor (TF) and can be efficiently translated. Hac1p forms a heterodimer with another bZIP transcription factor (TF), Gcn4p, to upregulate the expression of downstream genes, such as *BiP* and *PDI*, by directly binding to the promoter *cis*-elements, unfolded protein-response element 1 (UPRE1, CAGCGTG) and UPRE2 (TACGTG) [41,42].

In fission yeast, neither *HAC1* orthologs nor IRE1-dependent mRNA upregulation have been identified [43]. Instead, widespread IRE1-dependent mRNA downregulation was observed in response to ER stress. This is referred to as IRE1-dependent decay (RIDD), which was first reported in *Drosophila*. It was found that IRE1 cleaves mRNAs that encode secreted or membrane proteins that are located on ribosomes associated with the ER [44]. Degrading mRNAs encoding ER-destined proteins, rather than splicing a TF mRNA, appears to be the primary function of IRE1 in fission yeast [43]. It is important to note that the only exception to this role in fission yeast is the IRE1-dependent cleavage of *BiP1* mRNA in the 3′ UTR, which stabilizes the mRNA, resulting in increased BiP1 translation.

### 5.2. UPR in Mammals

While only one UPR sensor (IRE1) has been identified in yeast, mammalian systems have three types of UPR sensors, IRE1, PKR-like ER kinase (PERK) and membrane-associated TFs, such as activating transcription factor 6 (ATF6). Activation of all three arms of the UPR may be regulated by BiP. In their inactive state, the lumenal domains of IRE1, PERK and ATF6 are associated with BiP [45]. Upon ER stress, BiP is competed away from these ER stress sensors by an excess of unfolded/misfolded proteins, resulting in the oligomerization of IRE1 and PERK and the translocation of ATF6 to the Golgi [46–48]. On the other hand, misfolded proteins can also bind to and activate IRE1 directly [49,50].

IRE1 and its splicing mechanism are conserved in all eukaryotes analyzed to date. Mammals have two genes encoding IRE1 and a Hac1 ortholog, X-box binding protein 1 (XBP1). While splicing removes a large intron from *HAC1* in budding yeast, it only removes 26 b from *XBP1*, but that results in a frame shift and the translation of the spliced form, *XBP1*[51]. The spliced form, *XBP1*, encodes a bZIP TF that upregulates ER stress gene expression, such as *BiP* and *EDEM1*. RIDD has been identified in mammals as well.

ATF6 in mammalian cells is a type II ER membrane protein with a bZIP domain that faces the cytoplasmic side of the ER membrane and a Site-1-Protease (S1P) recognition site on the side facing the ER lumen. Upon ER stress, ATF6 translocates from ER to the Golgi aided by the ER export machinery, involving coat protein complex II (COPII). ATF6 is subject to sequential cleavages by a soluble lumenal protease, S1P, and a membrane associate, Site-2-Protease (S2P), resulting in the release of the cytoplasmic component of ATF6 [52,53]. This process is called regulated intramembrane proteolysis (RIP). The processed ATF6 is imported into the nucleus and functions as a TF to activate target genes expression [52]. Notably, ATF6 can also regulate the transcription of selective genes by chromatin modifications, such as the induction of *BiP* through methylation and acetylation of histone H4 in its promoter and the repression of cystic fibrosis transmembrane conductance regulator (CFTR) through DNA methylation and histone deacetylation [54,55].

Both XBP1 and ATF6 activate the UPR by directly binding to the promoters of ERQC/ERAD related genes. Three *cis*-elements capable of binding to ATF6 and/or XBP1 have been identified in mammals, ER stress responsive element (ERSE, CCAAT-N9-CCACG), ERSE-II (ATTGG-N-CCACG) and UPRE (TGACGTGG/A). While ERSE is responsible for the induction of ERQC components, the UPRE is mainly found in the promoters of ERAD-related genes [45]. Both ATF6 and XBP1 bind to the CCACG of the ERSE and the NF-Y TF complex binds to the CCAAT-box in the ERSE [51,56,57]. While ATF6 can also bind to the ERSE-II together with NF-Y, XBP1 binds to the ERSE-II and the UPRE in a NF-Y-independent manner [51,58].

In addition to transcriptional regulation, the UPR can also relieve ER stress at a translational level by the action of PERK in mammals [59]. PERK is a type I ER membrane protein with a cytoplasmic kinase domain. Upon ER stress, PERK phosphorylates the eukaryotic translational initiation factor 2 α-subunit (eIF2α), resulting in the attenuation of general translation, which reduces the throughput of new proteins in the stressed ER [59]. On the other hand, phosphorylation of eIF2α induces the translation of a small set of genes that contain short open reading frames in their 5′ UTRs, such as *ATF4*, another TF. ATF4 regulates the expression of a large set of genes involved in ERQC, amino acid metabolism, resistance to oxidative stress and PCD, such as *BiP*, *CHOP*, *GADD34* and *ATF3*[60–63].

### 5.3. UPR in Plants

Some of first reports of the UPR in plants derived from studies of zein mutants in maize. The mutants *floury-2* (*fl2*), *Mucronate* (*Mc*) and *defective endosperm B30* (De*-B30) encode defective storage proteins and displayed ER stress responses that were specific to the endosperm [64–66]. The *fl2* mutant produced a 24-kD α-zein with a defect in its signal peptide. As a consequence, the signal peptide on the defective storage protein was not cleaved, and the mutant zein accumulated as a membrane-anchored protein in the ER and in protein bodies [65,67]. The production of this chronically misfolded protein caused ER stress that resulted in the accumulation of BiP.

**Figure 3:**
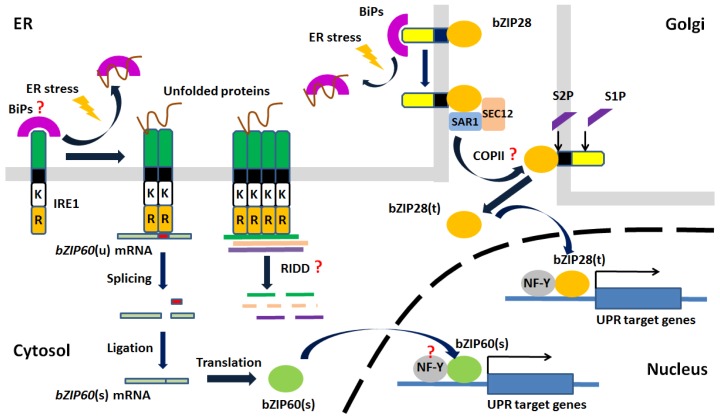
The unfolded protein response in *Arabidopsis. Arabidopsis* plants have two arms of the ER stress signaling pathway, an arm involving the proteolytic processing of membrane-associated basic leucine zipper domain (bZIP) transcription factors (TFs) and an arm involving RNA splicing factor, IRE1. The membrane-associated bZIP TFs, having a cytosol-facing TF domain, a single transmembrane domain and a canonical S1P site on their lumen-facing domain, are translocated to Golgi (by some means likely involving the COPII vesicle machinery; red question mark) and proteolytically processed by site-1 protease (S1P) and site-2 protease (S2P) in response to ER stress. The cytoplasmic components of the released TFs (e.g., bZIP28(t)) enter the nucleus and activate unfolded protein response (UPR) target genes together with CCAAT-box binding proteins composed of NF-Y subunits. Upon ER stress, IRE1 splices *bZIP60* mRNA, causing a frameshift leading to the synthesis of a TF without a transmembrane domain, but having acquired a nuclear targeting signal. The spliced form of bZIP60 (bZIP60(s)) is imported into the nucleus to activate UPR target genes. On the other hand, recent studies suggested that IRE1-dependent decay (RIDD) of specific mRNAs may also occur in *Arabidopsis* (red question mark). It is important to note that it is still unknown whether the activation of IRE1 and bZIP17/bZIP28 involve the disassociation of BiPs and whether bZIP60 functions as a TF together with NF-Y complexes (red question marks). K, kinase domain; R, RNase domain.

More recently, the components and the framework for the UPR signaling pathway in plants have come to light through studies in *Arabidopsis*. The ER stress signaling pathway in plants is reported to have two arms, an arm involving the proteolytic processing of membrane-associated bZIP TFs and an arm involving RNA splicing factor, IRE1 (Figure 3).

The membrane-associated bZIP TFs, bZIP17 and bZIP28, were recognized by their structural similarity to mammalian ATF6, having a cytosol-facing TF domain, a single transmembrane domain and a canonical S1P site in their lumen-facing domain. Both bZIP17 and bZIP28 are proteolytically activated by ER stress agents (DTT and TM) and by environmental stress conditions, such as heat and salt stress [68–70]. Upon ER stress, both TFs translocate from the ER to the Golgi, where they are subject to sequential cleavages by S1P and S2P. Then, the released cytoplasmic components relocate to the nucleus to activate ER stress response gene expression [68,69]. A recent study has shown that the interaction between bZIP28 and SAR1, a small GTPase involved in the formation of prebudding complexes for COPII-mediated relocation of cargo from the ER to the Golgi, is important for bZIP28 mobilization in response to stress [71]. It was also shown that bZIP28 activates ER stress response gene expression together with the NF-Y complex, consisting of At-NF-YA4, At-NF-YB3 and At-NF-YC2 [72].

Recently, another arm of the ER stress signaling pathway involving RNA splicing by IRE1 was identified in *Arabidopsis*, rice and maize [73–79]. In *Arabidopsis*, the target RNA, *bZIP60* mRNA, was identified by the distinctive structure of its IRE1 recognition site predicted by RNA folding programs [73]. *bZIP60* mRNA in an unspliced state encodes a protein similar to membrane-associated bZIP TFs, with a cytosol-facing TF domain, a single transmembrane domain, but no canonical S1P site on its lumen facing domain [80,81]. RNA splicing by IRE1 excises a 23b RNA segment, resulting in a mRNA encoding the same TF, now without a transmembrane domain, but having acquired a putative nuclear targeting signal. Knockout mutations in IRE1b or a single point mutation in a conserved base in one of the twin loops in bZIP60 mRNA prevents splicing and the upregulation of a bZIP60 target gene, *BINDING PROTEIN3* (*BiP3*) [73]. *Arabidopsis* has two full-length IRE1s, IRE1a and IRE1b. While IRE1b is mainly responsible for TM/DTT/Heat-induced bZIP60 splicing, IRE1a appears to play a primary role in SA/pathogen-induced *bZIP60* splicing [73,82]. However, IRE1a and IRE1b have overlapping functions, because *ire1a*, *ire1b* double mutants have more severe phenotypes than single mutants. On the other hand, a recent study showed that the downregulation of genes in response to ER stress is dependent on IRE1’s RNase activity, but not on OsbZIP50 (AtbZIP60 ortholog) in rice [83], suggesting that RIDD may also be active in these plants.

*cis*-elements analogous to mammalian ERSE, ERSE II and UPRE are also found in plant promoters, such as pERSE (CCAAT-N10-CACG), pUPRE (ATTGGTCCACGTCATC) and pUPRE-II (GATGACGCGTAC), and were found to be important for ER stress-induced gene expression [72,84–86].

### 5.4. Hypothetical Temporal Activation and Attenuation of the UPR

**Figure 4:**
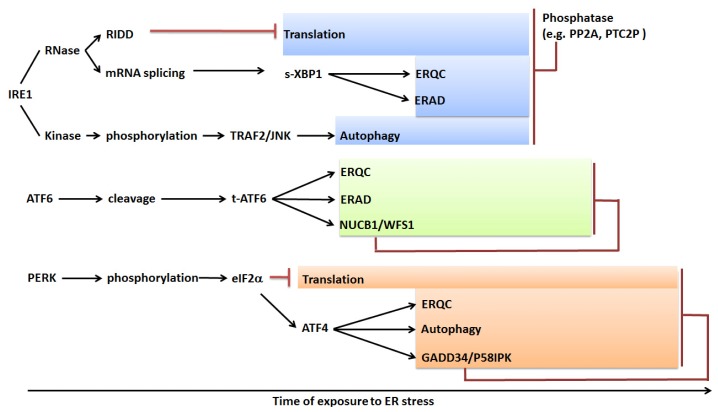
Hypothetical time course of the unfolded protein response in mammals. It has been proposed that the translation repression by PERK and IRE1-dependent decay (RIDD) occur early in response to ER stress, followed by ATF6-induced upregulation of ERQC and ERAD related genes. The upregulation of UPR target genes by XBP1 and PERK-ATF4 occurs next, since the activation of XBP1 and ATF4 themselves also require *de novo* protein synthesis. The responses in all three arms of the UPR undergo attenuation after the onset of stress, even under continuous stress conditions. While the IRE1 response attenuates quickly within eight hours through phosphatase-regulated IRE1 dephosphorylation, the ATF6 activation response lasts for about 20 h and, then, is suppressed by its own induced NUCB1 and WFS1. On the other hand, the PERK-mediated response persists even after 30 h and finally attenuates through eIF2α dephosphorylation regulated by GADD34/P58IPK, which are also induced by the PERK pathway itself.

Upon ER stress in mammalian cells, the activation of different arms of the UPR is phased. Within minutes of ER stress treatment, translation is repressed by PERK, and IRE1 begins to degrade ER-destined mRNAs. That is followed by transcriptional upregulation of two groups of stress-induced genes. The first group upregulated are the membrane-associated TFs, such as ATF6, which are activated by organelle-to-organelle movement and proteolytic processing. The second group includes PERK-ATF4 and XBP1, which are upregulated by the synthesis of ATF4 and XBP1 proteins. Considering that the latter is slower than proteolytic processing, ATF6 signals should precede those produced by XBP1 and PERK-ATF4 [87] (Figure 4).

The persistent upregulation of the UPR can be deleterious and the activities of all three arms of UPR in mammalian cells attenuate with time, even under continued stress, and as a consequence, the cell/organism survives [88]. IRE1 signaling attenuates quickly within eight hours after the onset of the response, followed by the attenuation of ATF6 arm, which lasts for about 20 hours. On the other hand, PERK signaling persists much longer, even more than 30 hours [89] (Figure 4).

IRE1 is negatively regulated by phosphatases, Ptc2p, PP2A and Dcr2, in yeast [90–92]. In mammals, the scaffold protein receptor for activated C-kinase 1 (RACK1) was found to recruit PP2A to IRE1 and dephosphorylate it [92]. After IRE1 activity attenuates, it has been reported in mammalian cells that the unspliced form of XBP1 can be translated and binds to the spliced form, XBP1, to induce the export and degradation of the XBP1 protein to the nucleus [93].

Upon ER stress, ATF6 in mammalian cells induces the expression of *Nucleobindin 1* (*NUCB1*) and *Wolfram syndrome 1* (*WFS1*), which can in turn deactivate ATF6 signaling by repression of the S1P-dependent cleavage of ATF6 and the enhancement of ATF6 ubiquitination and degradation [94,95]. Similarly, in response to ER stress, PERK-eIF2α-ATF4 induces the expression of *GADD34*, which encodes a PERK-inducible regulatory subunit of a protein phosphatase, PP1C, which in turn can dephosphorylate eIF2α. P58IPK is induced by ATF6 and XBP1 in response to ER stress and can also deactivate the PERK pathway by inhibiting eIF2α phosphorylation [96,97].

### 5.5. Evolutionary Relationship of UPR Components

The IRE1 arm of the UPR is thought to be the oldest arm of the signaling pathway, because it is found in all eukaryotes studied to date. The major role of IRE1 is transcriptional upregulation of ERQC/ERAD-related genes through the splicing of specific mRNAs in budding yeast (*HAC1*), mammals (*XBP1*) and plants (*bZIP60*). The effects of splicing differ for *HAC1*, *XBP1* and *bZIP60*. In budding yeast and mammals, the splicing of *HAC1* and *XBP1* mRNAs by IRE1 mainly affects their translation, while the splicing of *bZIP60* mRNA in plants may control the translation of the mRNA, but it also influences the subcellular location of the protein. Also, of note is the fact that there is only one full-length IRE1 in yeast and rice and two in *Arabidopsis* and mammals.

While there are no ATF6-like UPR sensors in yeast, there are two in *Arabidopsis* (bZIP17 and bZIP28) and several in mammals (including ATF6α, ATF6β, OASIS and CREBH), suggesting that this relative new arm of UPR may have evolved in multicellular organisms; PERK is only found in mammals, but not in yeast and plants. It is thought that PERK is an IRE1 homolog lacking a RNase domain, because it has been shown that the ER lumenal domains of IRE1 and PERK are interchangeable [98]. On the other hand, the cytoplasmic kinase domain of PERK may have originated from General Control Non-repressed 2 (GCN2), a protein kinase that phosphorylates eIF2α in response to amino acid starvation. GCN2 has been identified in all eukaryotes analyzed to date.

### 5.6. UPR-Induced ERAD and Autophagy

As an outcome of UPR signaling, ERAD and autophagy are activated, leading to the removal of unfolded and misfolded proteins from the ER. In mammals, IRE1 regulates ERAD through the XBP1-dependent transcriptional regulation of ERAD related genes, such as *EDEM1*[99]. IRE1 also regulates autophagy in a XBP1-independent manner, which helps to remove unfolded/misfolded proteins from ER [100,101]. Tumor necrosis factor receptor associated factor 2 (TRAF2) [102] and ASK1-interacting protein 1 (AIP1) have been shown to associate with IRE1 in animal cells [103]. Upon ER stress, phosphorylated IRE1 assembles a complex consisting of TRAF2, a protein kinase (IKK) and a MAPKKK, apoptosis signaling-regulating kinase factor 1 (ASK1) [102,104]. This complex further activates the c-Jun *N*-terminal kinase (JNK) through a MAPK cascade [101]. Upon ER stress, JNK-mediated BCL-2 phosphorylation promotes its dissociation from the pro-autophagy BH3 domain-containing protein, Beclin1, which mediates the interaction of other autophagy-related proteins with the pre-autophagosomal membrane, thereby inducing autophagy formation [105]. JNK may also induce autophagy formation by activating the autophagy regulator, ATG7, an E1 ubiquitin ligase-like enzyme [106]. Recent studies in mammals showed that the PERK-ATF4-CHOP pathway also induces autophagy through upregulation of the autophagy regulators, ATG6 and ATG8 [107], while ATF6 can induce ERAD through upregulation of ERAD components, such as *Derlin-3* (*Derl3*) [108].

In plants, there is no evidence for TRAF2 or JNK homologs. However, recent studies showed that an *ire1b* single mutant, but not *ire1a* or *bzip60* mutants, blocked ER stress-induced autophagy in *Arabidopsis*, suggesting that the regulation of autophagy by IRE1 is partially conserved [36].

### 5.7. UPR-Induced Programed Cell Death

If ER stress persists or if cells are subjected to severe stress conditions, the UPR transitions from a cell-saving to a cell-death process. In mammals, programmed cell death (PCD) can be classified as apoptosis, necrosis or autophagic cell death according to morphological criteria, and among these, apoptosis is the most common [109]. It has been proposed that all three UPR arms are involved in this process.

IRE1 was found in mammalian cells to directly associate with proteins involved in PCD pathway, including TRAF2 [102], AIP1 [103], the BCL-2 family members, BAX and BAK [110] and BAX inhibitor 1 (BI-1) [111]. Beside its role in ER stress-induced autophagy, ASK1-JNK can also transduce signals to the apoptosis pathway. JNK-mediated BCL-2 phosphorylation was shown to promote BCL-2’s dissociation from the pro-apoptotic BH3 domain-containing proteins, such as BAX, thereby, inducing apoptosis [105]. IRE1 can also activate caspase-12 through TRAF2 to induce apoptosis [112]. The BCL-2 family is a group of well characterized regulators of apoptosis, composed of both anti-apoptotic members, such as BCL-2 and pro-apoptotic members, such as BAX and BAK. BI-1 is a six transmembrane-containing protein that can suppress BAX-regulated PCD. It was found that BI-1 can bind to and suppress the activity of IRE1 in mouse [111]. However, how IRE1 regulates ER stress-induced PCD through these proteins is still unclear. On the other hand, recent studies showed that by artificially extending the period of IRE1 signaling, survival was reduced in yeast and mammalian cells, possibly through RIDD-induced apoptosis [113–115], suggesting that IRE1 may also regulate some PCD-related genes at a post-transcriptional level.

Upon ER stress, PERK upregulates *CHOP* in mammalian cells through the action of ATF4. CHOP is a TF that can control genes involved in apoptosis, such as the downregulation of the anti-apoptotic BCL-2 and upregulation of some BH3-only proteins, resulting in induction of apoptosis [116,117]. In mammals, ATF6 also mediates apoptosis by upregulation of a pro-apoptotic gene, *WW domain-binding protein 1* (*WBP1*) and downregulation of an anti-apoptotic gene, *Myeloid cell leukemia sequence 1* (*MCL-1*) [118]. On the other hand, ATF6 can also upregulate CHOP through direct binding to the ERSE of its promoter together with the CCAAT-box binding factor composed of NF-Y subunits.

In plants, there is no morphological equivalent of mammalian cell apoptosis, mainly because of the presence of the cell wall, which prevents blebbing from the plasma membrane and the engulfment of blebs from dying cells by phagocytes. Instead of that, plants have a specific type of PCD, vacuolar cell death, in which the cell contents are removed by vacuolization until the vacuole lyses [119]. The mechanism underlying ER stress-induced PCD is still largely unknown, as well as the PCD signal itself in plants. Relatives of the core components of mammalian apoptosis, such as BCL-2 family proteins, have not been identified in plants. However, the functional ortholog of mammalian caspase, metacaspase and the BI-1 ortholog, such as AtBI-1 in *Arabidopsis*, are found in plants. Metacaspases are believed to be the functional equivalents of mammalian caspases in yeast and plants, although they have different cleavage specificities [120]. There are nine metacaspase genes (*AtMC*) in the *Arabidopsis* genome, among them, *AtMC8* is highly upregulated by UV-C or H_2_O_2_-induced oxidative stress [121]. In protoplasts overexpressing *AtMC8* increased UV-C or H_2_O_2_-induced PCD, while knocking out *AtMC8* reduced PCD [121], suggesting that AtMC8 is a positive regulator of PCD signaling in plants.

In plants, AtBI-1 was identified as the homolog of mammalian anti-apoptotic gene, BI-1, by the finding that AtBI-1 can suppress Bax-induced cell death in yeast and plants [122–124]. The *atbi-1* mutant showed increased sensitivity to fumonisin (FB1) and heat shock-induced cell death [125], indicating that AtBI-1 is a negative regulator of PCD signaling in plants. On the other hand, *AtBI-1* is upregulated in response to TM in a bZIP60-dependent manner. *atbi-1* mutants showed increased sensitivity to TM-induced cell death, while overexpressing *AtBI-1* significantly reduced the sensitivity to TM, suggesting that AtBI-1 may also be involved in ER stress-induced PCD in plants [126], as well as the BI-1 in mammals. Although no BCL-2 ortholog has been identified in plants, Williams *et al*. found that a BCL-2-associated athanogene 7 (AtBAG-7), which belongs to a conserved protein family that functions as a co-chaperone in yeast and mammalian PCD pathways, directly interacts with BiP2 and its knockout showed increased sensitivity to TM-induced cell death in *Arabidopsis*[127].

## 6. Physiological Role of the ER Stress Response in Plants

In plants, ER stress responses are involved in both abiotic and biotic stress. Some mutants with deficiencies in UPR, ERQC or ERAD show increased sensitivity to various environmental stresses, while the others show dramatic vegetative or reproductive defects.

### 6.1. ER Stress Response and Abiotic Stress

Recent studies showed that the UPR is closely related to salt stress, heat stress and drought stress in plants. Both *s1p* and *bzip17* single mutants showed salt sensitive phenotypes [69] and overexpressing *bZIP60* showed more tolerance to salt stress in *Arabidopsis*[128], suggesting that both arms of the UPR are involved in the salt stress response. It was also found that heat can activate both two arms of the UPR in *Arabidopsis*. Che *et al*. observed that heat treatment increases the relocation of bZIP17 and bZIP28 to the nucleus [70], Deng *et al*. showed that heat can induce the *bZIP60* splicing [73] and Gao *et al*. found that the *bzip28* single mutant has a heat sensitive phenotype [129], suggesting that the UPR may play an important role in heat tolerance. Finally, overexpressing BiP in soybean or overexpressing a wheat CRT (Ta-CRT) in tobacco showed more tolerance to drought [130,131].

### 6.2. ER Stress Response and Biotic Stress

With regard to the role of the UPR in biotic stress, Moreno *et al*. found that the *ire1a* single mutant showed increased susceptibility to pathogen infection and is impaired in establishing systemic acquired resistance (SAR) [82]. It appears to be mainly due to a block in salicylic acid (SA) and pathogen-induced IRE1 mediated *bZIP60* splicing. Similarly, Tateda *et al*. showed that *NbbZIP60* silenced plants were more susceptible to *Pseudomonas cichorii* infection in *N. benthamiana*[132].

### 6.3. ER Stress Response and Plant Development

Beside environmental stresses, the UPR can also be activated under normal growth conditions or at specific developmental stages, when there is heavy protein secretion. Mutants with deficiencies in UPR, EQRC or ERAD can cause retarded growth phenotypes and even sterility.

Che *et al*. found that both *s1p* and *s2p* had impaired root growth, which could be complemented by the expression of the truncated and active form bZIP17 or bZIP28 [70]. S1P and S2p are two key proteases responsible for the cleavage and activation of bZIP17 and bZIP28 in Golgi. It has been proposed that bZIP17 and bZIP28 mediate root development through the brassinosteroid (BR) signaling pathway. Double *ire1a ire1b* mutants also exhibit short root phenotypes, suggesting that both two arms of UPR in plants mediate root development under normal condition [70,133].

The *Arabidopsis* GRP94 ortholog, *SHEPHERD* (*SHD*), which can be highly induced by TM or DTT treatment, was identified in a mutant showing expanded shoot apical meristems and floral meristems [72,134]. GRP94 is an ER chaperone acting in a way that is similar to BiP. It has been proposed that SHD may regulate meristem development through correct folding of CLAVATA proteins.

Some key components of ERQC and the UPR appear to be essential for other developmental processes, such as male or female gametophyte development and pollen elongation. For example, *Arabidopsis STT3A* and *STT3B* encode an essential subunit of the OST complex, which transfers oligosaccharides to newly synthesized ER proteins, and knocking out *STT3A* and *STT3B* affects both male and female gametophyte development [135]. On the other hand, SEC24, a component of COPII vesicles, is important for bZIP28 translocation from the ER to the Golgi, and knocking out *AtSEC24A* leads to a pollen structure defect in *Arabidopsis*[136]. Another example of the role of UPR in reproductive development is *thermosensitive male sterile 1* (*tms1*), which was identified as an *Arabidopsis* mutant showing significant reduction of male fertility at elevated temperature (30 °C) [137]. Further analysis indicated that knocking out TMS1 greatly affects pollen tube elongation at elevated temperature. *TMS1* encodes an ortholog of mammalian ERDJ3, which functions as a BiP co-chaperone.

## 7. Conclusions

Although having received considerable attention in recent years, the mechanisms underlying the plant ERQC, ERAD and ER-related autophagy and PCD are still largely unknown compared to yeast and mammalian systems. Some key components still need to be identified through further studies. The mechanisms of activation and attenuation of the UPR in plants and how the UPR promotes either cell survival or cell death need to be elucidated. The UPR appears to have important physiological roles in plants, and understanding those roles could be of potential benefit to plant improvement.
